# The impact of unrecognized autoimmune rheumatic diseases on the incidence of preeclampsia and fetal growth restriction: a longitudinal cohort study

**DOI:** 10.1186/s12884-016-1076-8

**Published:** 2016-10-18

**Authors:** Arsenio Spinillo, Fausta Beneventi, Elena Locatelli, Vèronique Ramoni, Roberto Caporali, Claudia Alpini, Giulia Albonico, Chiara Cavagnoli, Carlomaurizio Montecucco

**Affiliations:** 1 Department of Obstetrics and Gynecology, University of Pavia, IRCCS Policlinico San Matteo Foundation, P.le Golgi 19, 27100 Pavia, Italy; 2 Department of Rheumatology, University of Pavia, IRCCS Policlinico San Matteo Foundation, Pavia, Italy; 3 Laboratory Medicine, University of Pavia, IRCCS Policlinico San Matteo Foundation, Pavia, Italy

**Keywords:** Autoimmune rheumatic diseases, Preeclampsia: Fetal growth restriction, Pregnancy, Connective tissue diseases

## Abstract

**Background:**

The burden of pregnancy complications associated with well defined, already established systemic rheumatic diseases preexisting pregnancy such as rheumatoid arthritis, systemic lupus erythematosus or scleroderma is well known. Systemic rheumatic diseases are characterized by a long natural history with few symptoms, an undifferentiated picture or a remitting course making difficult a timely diagnosis. It has been suggested that screening measures for these diseases could be useful but the impact of unrecognized systemic rheumatic disorders on pregnancy outcome is unknown. The objective of the study was to evaluate the impact of previously unrecognized systemic autoimmune rheumatic on the incidence of preeclampsia and fetal growth restriction (FGR).

**Methods:**

A longitudinal cohort-study with enrolment during the first trimester of pregnancy of women attending routine antenatal care using a two-step approach with a self-reported questionnaire, autoantibody detection and clinical evaluation of antibody-positive subjects. The incidence of FGR and preeclampsia in subjects with newly diagnosed rheumatic diseases was compared to that of selected negative controls adjusting for potential confounders by logistic regression analysis.

**Results:**

The prevalence of previously unrecognized systemic rheumatic diseases was 0.4 % for rheumatoid arthritis (19/5232), 0.25 % (13/5232) for systemic lupus erythematosus, 0.31 % (16/5232) for Sjögren’s syndrome, 0.3 % for primary antiphospholipid syndrome (14/5232) and 0.11 % (6/5232) for other miscellaneous diseases. Undifferentiated connective tissue disease was diagnosed in an additional 131 subjects (2.5 %). The incidence of either FGR or preeclampsia was 6.1 % (36/594) among controls and 25.3 % (50/198) in subjects with unrecognized rheumatic diseases (excess incidence = 3.9 % (95 % CI = 2.6–9.6) or 34 % (95 % CI = 22–44) of all cases of FGR/preeclampsia). The incidence of small for gestational age infant (SGA) was higher among subjects with unrecognized rheumatic diseases (41/198 as compared to 46/594; adjOdds Ratio = 3.1, 95 % CI =1.96–4.95) than in controls. The excess incidence associated with unrecognized rheumatic diseases was 2.7 % (95 % CI = 1.5–4) or 25 % (95 % CI = 12.8–34.8) of all SGA cases.

**Conclusions:**

Unrecognized autoimmune systemic rheumatic disorders are associated with a significant proportion of preeclampsia and fetal growth failure, suggesting that their role in the etiology of adverse pregnancy outcome is probably undervalued.

## Background

The major systemic autoimmune rheumatic diseases, including rheumatoid arthritis (RA), systemic lupus erythematous (SLE), Sjögren’s syndrome, and other connective tissue diseases are relatively common in the general population, with a lifetime risk of 8.4 % among women [1]. It has been estimated that approximately one in 12 women will develop a definite inflammatory rheumatic disease during her lifetime [1]. In addition, establishing and emerging data suggest that the natural history of RA, SLE and other autoimmune rheumatic disease encompasses a so-called preclinical disease phase lasting from months to several years and characterized by little or no clinical findings and by the presence of detectable autoimmune antibodies [2, 3]. These data suggest that autoantibodies and autoimmune rheumatic diseases at various stages of development, often unrecognized or undiagnosed, are much more common in the general population than previously recognized [3, 4]. Systemic rheumatic disorders or the preclinical stages of these diseases are not entirely benign and have been associated with increased risks of subclinical atherosclerosis, ischemic heart disease and lung diseases [2, 5]. In addition, several studies have shown that higher than expected rates of reproductive failures, fetal growth restriction and preeclampsia occur before the clinical occurrence or the diagnosis of rheumatic diseases [6, 7], suggesting a potential causal role of unrecognized or undiagnosed autoimmune disorders in the occurrence of pregnancy complications. The purpose of this study was to evaluate the prevalence of previously unrecognized autoimmune rheumatic disorders during pregnancy and to measure their impact on the incidence of fetal growth failure and preeclampsia.

## Methods

Subjects for the study were recruited among unselected pregnant women who obtained antenatal care at our Department during the first trimester of pregnancy. As this was a cohort pilot study involving only a few members of staff, we restricted the enrolment to all women attending the clinic for antenatal care each Monday during a 6-year period (May 2009 to June 2014). The study was approved by the local ethics committee of our Department (Procedure n.20110034530). Enrolment criteria included: a) singleton pregnancy; b) antenatal care and delivery at our Department; c) fluency in the Italian language; d) no previous diagnosis or treatment of connective tissue diseases; e) absence of fetal malformations or chromosomal anomalies. The characteristics of the study and the validation of the methods used have been already reported elsewhere [8]. Briefly, after informed consent and before the medical evaluation, each woman was asked to complete a screening questionnaire including any connective tissue disorder symptoms (Table 1). Women who answered positively to one or more of the questions were tested for the presence of circulating autoantibodies, including antinuclear antibody (ANA), anti-double-stranded DNA, anti-extractable nuclear antigen (ENA), anticardiolipin antibody, anti-β2-glycoprotein I antibodies (aβ2GPI) and lupus anticoagulant, according to standardized methods, as previously described [9]. The ANA test was considered positive at a titer ≥1:80. To ensure random sampling, the first three subjects with negative responses to all the items in the questionnaire after each index case diagnosed with a rheumatic disease (major or undifferentiated connective tissue diseases) and willing to participate in the study were tested for autoantibodies and served as the control. Cases and controls were referred to the rheumatology unit of our hospital for further clinical assessment including a careful history and a physical examination. Rheumatologists were unaware of the results of the questionnaires. Rheumatoid factor and anti-citrullinated peptide antibodies were not included in the screening autoantibody profile, but were tested only in patients with arthritis after the rheumatological evaluation. Rheumatic diseases were classified according to widely used criteria for undifferentiated connective tissue disease (UCTD) [10], RA [11], SLE [12], anti-phospholipid syndrome (APS) [13], Sjögren’s syndrome [14], systemic sclerosis [15], polymyositis/dermatomyositis [16] and mixed connective tissue disease [17]. Patients with suspected rheumatic disease (symptoms plus autoantibodies) but not fulfilling the abovementioned criteria were classified as the no criteria for diagnosis group. Monthly rheumatological clinical assessment during pregnancy was carried out in subjects with major or undifferentiated connective tissue diseases and in those without criteria for a definite diagnosis.

**Table 1:** Ten-item questionnaire administered

1. Have you ever had generalised or localized reddening of your skin after exposure to sunlight?
2. Have you ever had an obvious or prominent rash on your cheeks or nose?
3. Do your hands or your feet white in the cold and then blue or pink?
4. a. Have you ever had painful and swollen joints? b. Do you suffer from stiffness lasting one hour or more in the morning?
5. Have you ever had pericarditis or pleuritis?
6. Do you have a dry mouth?
7. Do you feel like you have sand in your eyes?
8. Have you ever had painful white mouth ulcers?
9. Have you ever had thrombophlebitis?
10. Have you had two or more miscarriages or stillbirths?

After Ist trimester enrolment, gestational age was confirmed by ultrasound, and cases and controls were followed-up with monthly obstetric clinical and ultrasonographic evaluations. The mean uterine artery pulsatility index (PI) in the first and second trimester was evaluated according to standard methods [18]. Pulsatility indices of uterine or umbilical arteries were considered abnormal when the values were higher than the 95^th^ percentile of reference curves [18]. FGR was diagnosed when the abdominal fetal circumference at ultrasonographic examination fell below the 10^th^ percentile of our local reference curves, confirmed on at least two consecutive measurements taken 2 weeks apart after the standard US obtained at 18–22 weeks of pregnancy, and PI of umbilical artery was higher than the 95^th^ percentile of reference curves signaling a reduced perfusion of fetal placental unit. Preeclampsia was diagnosed according to standard criteria [19]. Small for gestational age infants were diagnosed when birth weight was below the 10^th^ percentile of the Italian population [20].

Statistical analyses were carried out with one-way analysis of variance and the Bonferroni post-hoc test to compare continuous variables between the groups studied. Categorical variables were compared by Pearson’s χ^2^. Partitioning of χ^2^ statistics with the Bonferroni correction for multiple comparisons was used to evaluate the statistical significance of pairwise comparisons in two × K tables. Associations between the diagnostic category of the rheumatic disorder and pregnancy outcomes were evaluated using logistic regression analysis by computing odds ratios and 95 % confidence intervals, adjusting for potential confounders (Stata 12.0 for Windows. (StataCorp LP, College Station, TX, USA).

Logistic models included complications of pregnancy (preeclampsia, FGR, SGA) as outcome variables and nulliparity (yes, no), first trimester smoking (yes, no), previous history of low birthweight (<2500 g) infant, chronic hypertension (yes, no) and the diagnostic category of the autoimmune rheumatic disease (major rheumatic disease, undifferentiated connective tissue disease, no criteria for diagnosis) as explanatory variables. Logistic regression was also used to compute the percent excess incidence (percent population attributable risk) and the percent fraction of outcome (percent population attributable fraction) associated with autoimmune rheumatic diseases [21].

## Results

Out of the 5451 eligible subjects enrolled during the period of the study, 5232 (96 %) gave their consent and completed the questionnaire. The rate of positivity to one or more questions was 9.8 % (511/5232). Of the 511 subjects with a positive questionnaire, 349 (68.3 %) tested positive for autoantibodies and were sent to the rheumatology unit for evaluation. The prevalence of the different rheumatic diseases was 0.4 % for RA (19/5232), 0.25 % (13/5232) for SLE, 0.31 % for Sjögren’s syndrome (16/5232), 0.3 % for primary APS (14/5232) and 0.11 % for other miscellaneous causes (two subjects with systemic sclerosis, one with mixed connective tissue disease one with Wegener syndrome and one with monoarticular arthritis). The overall prevalence of major connective tissue disease was 1.3 % (68/5232). UCTD was the most common unrecognized rheumatologic disorder and was diagnosed in 2.5 % (131/5232) of subjects. Finally, 150 out of the 349 subjects with symptoms and autoantibodies (43 %) had insufficient criteria for a diagnosis of a definite rheumatic disease.

Table 2 reports the distribution of symptoms and autoimmune antibodies in the groups studied. More than 50 % of women with UCTD or a with major rheumatic disease reported three or more symptoms; on the other hand, 5.4 % of controls tested positive for antinuclear antibodies at a titer of 1:80 or more. Photosensitivity and Raynaud’s phenomenon were the most common symptoms among subjects diagnosed with UCTD (73/131 and 92/131) and those without a definite diagnosis (85/150 and 78/150, respectively). Of the 150 subjects without a definite diagnosis, 116 ANA (77.3 %) positive subjects had a clinical picture resembling UCTD but with symptoms lasting less than 3 years, whereas the remaining 34 (22.7 %) had an incomplete picture of a major rheumatic disease.

**Table 2:** Number of positive questionnaire answers and autoantibodies among controls and subjects with undifferentiated connective tissue disease, major rheumatic diseases and no criteria for diagnosis

	Controls *n* = 597	UCTD *n* = 131	Major rheumatic diseases *n* = 68	No criteria for diagnosis *n* = 150
N. of items	N (%)	N (%)	N (%)	N (%)
1	**-**	8 (61.1)	8 (11.8)	**-**
2	**-**	54 (41.2)	24 (35.3)	87 (58)
>2	**-**	69 (52.7)	36 (52.9)	63 (42)
ANA titre				
1:80	24 (4)	42 (32.1)	13 (19.1)	82 (54.6)
1:160	8 (1.3)	46 (35.1)	16 (23.5)	26 (17.3)
> 1:160		43 (32.8)	26 (38.2)	11 (7.3)
dsDNA	0	5 (3.8)	8 (11.7)	2 (1.3)
ENA	0	17 (12.9)	22 (32.4)	0
aCLIgG	0	6 (4.6)	10 (14.7)	9 (6)
aCL IgM	1 (0.16)	14 (10.7)	11 (16.2)	22 (14.6)
beta2GP1IgG	0	4 (3.05)	8 (11.7)	8 (5.3)
LAC	0	1 (0.76)	9 (13.2)	0

*UCTD* undifferentiated connective tissue disease, *ANA* antinuclear antibodies, *dsDNA* anti-double stranded DNA, *ENA* extractable nuclear antigen antibodies, *aCL* anticardiolipin autoantibodies, *beta2GP1* beta2glicoprotein1, *LAC* lupus anticoagulant

After the first trimester of pregnancy, corticosteroids and/or hydroxychloroquine were given to 23 (17.6 %) women with UCTD and to 20 (29.4 %) with a definite connective tissue disease. After a definite rheumatological diagnosis, low-molecular dose heparin and/or aspirin were administered in 18 (26.5 %) and 42 (61.8 %) of subjects with major rheumatic diseases and 15 (11.5 %) and 48 (36.6 %) of those with UCTD, respectively. Overall, among the 199 women with a definite rheumatic disease, heparin was given to 26 (13.1 %) subjects with primary or secondary APS and to 7 (3.5 %) women with a previous thrombotic events.

Infants born to subjects with rheumatic symptoms, irrespective of diagnosis, had a lower gestational age and birthweight than controls (Table 3). In the post-hoc analysis, and after Bonferroni correction, gestational age and birthweight among subjects with a major rheumatic disease were lower when compared to the UCTD group (*p* = 0.05 for both comparisons) and to subjects without a definite diagnosis (*p* <0.001 and *p* = 0.012 for gestational age and birthweight, respectively). Nulliparity was more common among controls than in any category of rheumatologic disorder (*p* <0.05 for all comparisons), whereas a previous low-birthweight infant was more common among subjects with a major rheumatic disease than in controls.

**Table 3:** Main demographic variables autoantibodies among controls and subjects with undifferentiated connective tissue disease (UCTD), major rheumatic diseases and no criteria for diagnosis

	Controls *n* = 597	UCTD *n* = 131	Major rheumatic diseases *n* = 68	No criteria for diagnosis *n* = 150	*p*
	mean (SD)	mean (SD)	mean (SD)	mean (SD)	
Maternal age (years)	33.2 (4.5)	33.6 (4.9)	33.6 (4.9)	33.5 (4.5)	0.65
Body mass index (Kg\m2)	23 (3.4)	22.8 (3.7)	22.6 (3.1)	22.7 (3.1)	0.66
Gestational age at entry (weeks)	13.1 (1.3)	13 (1.4)	12.9 (1.2)	13.2 (1.4)	0.7
Gestational age at birth (weeks)	39 (1.9)	38.4 (2.1)	37.7 (2.9)	38.9 (1.99)	<.001
Birth Weight (gr)	3242 (506)	3050 (607)	2844 (664.77)	3086 (492)	<.001
	N (%)	N (%)	N (%)	N (%)	
Caucasian	573 (96)	127 (97)	64 (94)	141 (94)	0.8
Black/African	21 (3.5)	3 (2.3)	4 (6)	8 (5.3)	
Asian	3 (0.5)	1 (0.7)		1 (0.7)	
Education (years)					
≤8	98 (16.4)	19 (14.5)	10 (14.7)	22 (14.7)	0.9
8–13	318 (53.3)	75 (57.3)	37 (54.4)	76 (50.7)	
>13	181 (30.3)	37 (28.2)	21 (30.9)	52 (34.7)	
Nulliparous	417 (69.8)	80 (61.1)	39 (57.3)	96 (64)	<.001
First trimester smoking	83 (13.9)	26 (19.8)	14 (20.6)	30 (20)	0.1
Previous low birth weight infant (<2500 gm)	4 (0.67)	4 (3.1)	3 (4.4)	4 (2.6)	0.03
Chronic hypertension	7 (1.2)	6 (4.6)	2 (2.9)	1 (0.6)	0.06

*SD* standard deviation

*p* values obtained by one-way anova or chi-square test

Increased placental vascular resistance, as suggested by increased pulsatility indices in the first and second trimester uterine artery and third trimester umbilical artery, were higher in all the categories of rheumatic disorders, including subjects without a definite diagnosis, than in controls (*p* <0.01 for all comparisons in the post-hoc analysis) (Table 4). Umbilical artery PI was also higher among major rheumatic diseases when compared to either UCTD (*p* = 0.009) or to subjects without a definite diagnosis (*p* = 0.017). These results were also confirmed in the categorical analysis since the rates of first and second trimester uterine artery bilateral notches and third trimester umbilical artery PI > 95^th^ percentile were higher among all categories of rheumatic disorders than in controls (*p* < 0.01 for all comparisons). In the partition of the chi-square analysis, compared to the negative controls, the rates of preeclampsia or FGR were more common among all categories of rheumatic disorders, including subjects without a definite diagnosis (*p* <0.01 for all comparisons). Finally, the rates of SGA infants were higher among patients with major rheumatic diseases (*p* <0.001) and UCTD (*p* = 0.007) than in controls.

**Table 4:** Maternal and fetal Doppler velocimetry, and main obstetric outcomes among controls and subjects with undifferentiated connective tissue disease (UCTD), major rheumatic diseases and no criteria for diagnosis

	Controls *n* = 597	UCTD *n* = 131	Major rheumatic diseases *n* = 68	No criteria for diagnosis *n* = 150	*p*
	mean (SD)	mean (SD)	mean (SD)	mean (SD)	
I st trimester Uterine artery PI	1.16 (0.3)	1.64 (0.3)	1.80 (0.4)	1.37 (0.4)	<.001
II nd trimester Uterine artery PI	0.87 (0.4)	1.29 (0.2)	1.33 (0.3)	1.00 (0.3)	<.001
III rd trimester Umbilical Artery PI	0.89 (0.1)	0.95 (0.2)	1.03 (0.3)	0.95 (0.20)	<.001
	N (%)	N (%)	N (%)	N (%)	
Uterine artery bilateral notch					
Ist trimester	23 (3.8)	33 (25.2)	30 (44.1)	14 (9.3)	<.001
IInd trimester	14 (2.3)	15 (11.5)	18 (26.5)	9 (6)	<.001
Umbilical artery PI >95° percentile	25 (4.2)	21 (16)	18 (26.5)	24 (16)	<.001
Miscarriage	2 (0.3)			1 (0.6)	0.6
Stillbirth	1 (0.2)		1 (1.5)		0.3
Gestational diabetes	13 (2.2)	4 (3.1)	4 (5.8)	6 (4.0)	0.35
Small for gestational age	46 (7.7)	23 (17.5)	18 (26.5)	17 (11.3)	<.001
Fetal growth restriction (FGR)	25 (4.2)	21 (16)	18 (26.5)	20 (13.3)	<.001
Preeclampsia	19 (3.2)	18 (13.7)	15 (22.1)	9 (6)	<.001
Preclampsia or FGR	36 (6)	30 (22.9)	20 (29.4)	22 (14.6)	<.001
Delivery less than 34 weeks	17 (2.8)	6 (4.6)	5 (7.4)	4 (2.6)	0.2
Cesarean section	183 (30.6)	58 (44.3)	34 (50)	46 (30.6)	0.001

*SD* standard deviation, *PI* pulsatility index

*p* values obtained by one-way anova or chi-square test

Table 5 reports the results of the logistic regression analysis. Putative risk factors for preeclampsia and/or fetal growth failure that differed among cases and controls at *p* ≤ 0.1 level (nulliparity, first trimester smoking, previous low birthweight infant and chronic hypertension) were inserted in the model as confounders. The likelihood of either FGR or preeclampsia was five times higher among subjects with defined rheumatic disorders, either UCTD or a major systemic disease, than in controls. The odds ratio of these two outcomes was also higher in subjects with a definite diagnosis of a rheumatic disease than in those with insufficient criteria for a diagnosis (*p* = 0.028). Overall, unrecognized rheumatic diseases, major or UCTD, were associated with an excess incidence of 3.9 cases per 100 subjects (95 % CI = 2.6–9.6), or 34 % (95 % CI = 22–44) of all cases of either preeclampsia or FGR.

**Table 5:** Crude incidence, odds ratio, population attributable risk and population attributable fraction of preeclampsia and/or fetal growth restriction (FGR) in the population studied after excluding subjects with spontaneous abortions

*Preeclampsia*	Crude incidence (%) 95 % CI	Odds ratio (95 % CI)	PAR (%) 95 % CI	PAF (95 % CI)
Controls/overall incidence* (*n* = 594)	3.2 (1.9–5)	Reference	6.5 (5.1–8.1)*	
Major rheumatic diseases (*n* = 67)	26.9 (16.8–39.1)	9.2 (4.3–19.5)	1.4 (0.6–2.1)	0.22 (0.09–0.33)
UCTD (*n* = 131)	13.7 (8.4–20.8)	4.6 (2.3–9.2)	1.4 (0.6–2.1)	0.21 (0.1–0.31)
Overall rheumatic diseases (*n* = 198)	16.7 (11.8–22.6)	6 (3.3–10.9)	2.8 (1.7–3.9)	0.43 (0.26–0.56)
No criteria for diagnosis (*n* = 150)	6 (2.8–11.1)	2 (0.9–4.6)	0.05 (−0.01–1.1)	0.07 (−0.03–0.17)
*Fetal growth restriction (FGR)*				
Controls/overall incidence*	4.2 (2.8–6.1)	Reference	8.8 (7.2–10.8)*	
Major rheumatic diseases	26.9 (16.8–39.1)	8.8 (4.4–17.3)	1.6 (0.9–2.4)	0.18 (0.1–0.26)
UCTD	16 (10.2–23.5)	4.1 (2.2–7.8)	1.5 (0.06–2.4)	0.17 (0.07–0.26)
Overall rheumatic diseases	19.7 (14.4–25.9)	5.5 (3.2–9.5)	3.1 (1.9–4.3)	0.36 (0.22–0.47)
No criteria for diagnosis	13.4 (8.4–20)	3.6 (1.9–6.7)	1.4 (0.05–2.3)	0.16 (0.06–0.26)
*Preeclampsia or FGR*				
Controls/overall incidence*	6.1 (4.3–8.3)	Reference	11.5 (9.6–13.5)*	
Major rheumatic diseases	29.9 (19.3–42.3)	6.8 (3.6–12.9)	1.7 (0.09–2.5)	0.15 (0.08–0.21)
UCTD	22.9 (16–31.1)	4.4 (2.5–7.5)	2.2 (1.1–3.2)	0.19 (0.1–0.27)
Overall rheumatic diseases	25.3 (19.4–31.9)	5.1 (3.2–8.3)	3.9 (2.6–9.6)	0.34 (0.22–0.44)
No criteria for diagnosis	14.8 (9.5–21.5)	2.7 (1.5–4.8)	1.4 (0.4–2.3)	0.1 (0.04–0.2)

*UCTD* undifferentiated connective tissue disease

Odds ratios (OR), population attributable risk (PAR) and population attributable fraction obtained by logistic regression analysis containing preeclampsia and/or FGR as outcomes and category of rheumatic disorders (controls, major rheumatic diseases, UCTD, no criteria for diagnosis), nulliparity (yes, no), first trimester smoking (yes, no), chronic hypertension (yes, no) and previous low birthweight (<2500 g) infant (yes, no) as explanatory variables. The analysis was carried out in 941 viable pregnancies

*Overall incidence

The overall adjusted prevalence of SGA in the population studied was 11.1 % (95 % CI = 9.2–13.2) (104/941). Major rheumatic diseases and UCTD were associated with an excess risk of SGA of 1.4 % (95 % CI = 0.6–2.1) and 1.3 % (95 % CI = 0.4–2.3), and with attributable fractions of 12.3 % (95 % CI = 5.2–18.8) and 12.5 % (95 % CI = 3.5–20.4), respectively. Overall, previously unrecognized rheumatic diseases, either major or UCTD, were associated with a significantly increased risk of SGA (OR = 3.1, 95 % CI = 1.96–4.95) and with an excess risk of 2.7 cases (95 % CI = 1.5–4) or 25 % (95 % CI = 12.8–34.8) of all SGA cases.

## Discussion

The results of this study have shown that unrecognized or undiagnosed autoimmune rheumatic disorders are rather common during the first trimester of pregnancy and are associated with a significant number of preeclampsia and fetal growth restriction cases. In particular, the 1.3 % rate of definite rheumatic diseases detected during the first trimester of pregnancy was responsible for 15 % of all cases of preeclampsia or IUGR and for 12 % of all cases of SGA. After the inclusion of the 2.5 % rate of UCTD detected during the first trimester, unrecognized rheumatic disorders were responsible for up to 25 % of SGA and up to 34 % of preeclampsia/IUGR cases in our population. The strengths of this study include the prospective design, the methods used and the number of subjects recruited. Previous retrospective studies have suggested that a clinical history of reproductive failure, preeclampsia, prematurity or FGR increases the subsequent risk of a diagnosis of rheumatic disease [22]. This association has prompted several authors to postulate a causal association between adverse reproductive events and subsequent occurrence of rheumatic diseases such as RA or SLE [23]. Although the potential interference of pregnancy-associated hormonal and metabolic modifications on the subsequent occurrence of rheumatic diseases cannot be entirely excluded, other retrospective studies suggest that a poor reproductive outcome is rather a comorbidity of the long preclinical phase of some rheumatic diseases rather than a true causative factor [3, 6, 7]. Our longitudinal data show that adverse obstetric events are common, not only among women with unrecognized rheumatic diseases with a definite diagnosis, but also among undifferentiated or even “early” rheumatic disorders which, without screening measures, would have been undetected.

Potential selection biases are the main limitations of the study. We used a two-step screening approach including a self-administered questionnaire and subsequent autoantibody detection, which in a pilot study demonstrated excellent detection rates of rheumatic disease [8]. In addition, the validity of a similar approach has been extensively confirmed in the diagnosis of RA, SLE and Sjögren’s syndrome both in Europe and in other countries [11–16]. The rate of false negatives using this method is very low [8]; thus, the prevalence rates detected in our study should not be biased by underdiagnosis. In addition, to avoid overdiagnosis, we used standard definitions for the diagnosis of RA, SLE, Sjögren’s syndrome, systemic sclerosis, APS and UCTD and rheumatological clinical assessments were repeated monthly during pregnancy. The overall rate of definite rheumatic diseases and UCTD in our study is consistent with the 5 % rate of major rheumatic diseases reported in the general population [1, 3], however, since recruitment took place in a public hospital, we cannot exclude a potential selection bias based on low socioeconomic status. Definite rheumatic diseases such as RA, SLE or Sjögren’s syndrome can be unrecognized or undiagnosed for long periods of time because the symptoms and signs are not specific and a precise diagnosis can be difficult to achieve [2–4]. Moreover, the natural history of these disorders follows a pattern of progression lasting from months to years, from a preclinical non-diagnostic phase to overt clinical disease [2, 3, 24]. Establishing the exact moment of transition from the preclinical to clinical phases is difficult; thus, in the general population, rheumatic diseases can be detected at various stages of development [24]. For these reasons, the 1.3 % rate of well-defined rheumatic disease in our population was not surprising. UCTD, defined as the presence of at least one clinical manifestation of a major rheumatic disease lasting at least 3 years and a positive ANA result, was the most frequent rheumatic disorder detected in our subjects. Although the risk of preeclampsia and fetal growth restriction associated with UCTD was lower than that associated with major rheumatic diseases, the burden of pregnancy complications associated with these two disorders was similar. The group of subjects with UCTD probably includes either women in the preclinical stage of a major rheumatic disease or women with a stable mild rheumatic disorder [25]. Whatever the composition of this group of rheumatic disorders, our results confirm preliminary data showing that UCTD is associated with an increased risk of adverse pregnancy outcomes [26]. According to Mosca et al., [27], the group of subjects with no criteria for a definite diagnosis of a rheumatic disease probably contains subjects in the early phase of stable UCTD as well as subjects at an early phase or in a true preclinical stage of a major rheumatic disease. Although, in our study, the impact of this condition on the incidence of FGR or preeclampsia was considerably lower than that of either UCTD or major rheumatic diseases, the risk of adverse pregnancy outcomes was higher in these subjects than in negative controls.

Inadequate trophoblast invasion of the spiral arteries with a subsequent defective establishment of maternal-fetal vascularization and oxygen and nutrient exchange is considered the probable mechanism underlying the adverse effect of rheumatic disorders on pregnancy outcomes [27]. A direct effect of maternal autoantibodies on trophoblast invasiveness as well as maternal proinflammatory status and endothelial dysfunction seem to mediate defective placentation among subjects with rheumatic disorders [27]. The results of our study confirm these suggestions. In fact, we found increased second trimester pulsatility indices and bilateral notching of uterine arteries in all categories of rheumatic disorders compared to controls. These Doppler findings are associated with inadequate trophoblast invasion of myometrial spiral arteries, leading to an increased risk of preeclampsia and FGR [23, 27]. A vascular mechanism could also be postulated for the adverse effect of preclinical rheumatic diseases on pregnancy outcome. In fact, it has been demonstrated that autoantibodies related to pre-disease stages could increase the risk of peripheral vascular damage, leading to accelerated atherosclerosis and cardiovascular disease [5].

The rate of unrecognized rheumatic disorders in the general population could be affected by the incidence of autoimmune diseases in the population and by the time and the frequency of contact with the health care system. For these reasons, our results cannot be generalized to other populations. However, given their long and often unpredictable natural history, it is likely that in the general population of countries with a high incidence of rheumatic disorders, a significant proportion of subjects in the preclinical stage of these diseases can go undetected for a long period of time. The identification of this group of subjects early during pregnancy could lead to a timely treatment of rheumatic disorders and also to a prevention of pregnancy complications [28].

## Conclusions

Our data indicate that a first trimester detailed history on symptoms suggesting a systemic connective tissue disease can be useful in the early detection of a previously undiagnosed systemic rheumatic disease. Screening measures for rheumatic disorders have the potential to improve both disease outcomes and comorbidities such as cardiovascular disease and adverse reproductive consequences [3, 29]. However, factors regulating the performance of a screening approach such as tests to be used, the population to be tested, the magnitude of net benefits and the harms associated with screening for rheumatic disorders are still uncertain [2–4]. Future studies may clarify if screening programs are applicable to populations at risk and whether the identification of subjects with rheumatic disorders at various stages of the disease could translate to a better prognosis and the prevention of associated comorbidities such as pregnancy complications.

## Abbreviations

ANA: Antinuclear antibody (ANA)
APS: Anti-phospholipid syndrome
ENA: Anti-extractable nuclear antigen
FGR: Fetal growth retsriction
PI: Pulsatility index
RA: Rheumatoid arthritis
SGA: Small for gestational age
SLE: Systemic lupus erythematous
UCTD: Uundifferentiated connective tissue disease
